# Cost-effectiveness of patient navigation programs for stroke patients–A systematic review

**DOI:** 10.1371/journal.pone.0258582

**Published:** 2021-10-15

**Authors:** Benjamin Kass, Christina Dornquast, Andreas Meisel, Christine Holmberg, Nina Rieckmann, Thomas Reinhold

**Affiliations:** 1 Institute for Social Medicine, Epidemiology and Health Economics, Charité –Universitätsmedizin Berlin, Corporate Member of Freie Universität Berlin, Humboldt-Universität zu Berlin, and Berlin Institute of Health, Berlin, Germany; 2 Department of Neurology with Experimental Neurology, Center for Stroke Research Berlin, Neurocure Clinical Research Center, Charité –Universitätsmedizin Berlin, Berlin, Germany; 3 Institute of Public Health, Charité –Universitätsmedizin Berlin, Corporate Member of Freie Universität Berlin, Humboldt-Universität zu Berlin, and Berlin Institute of Health, Berlin, Germany; 4 Institute of Social Medicine and Epidemiology, Faculty of Health Sciences Brandenburg, Brandenburg Medical School Theodor Fontane, Brandenburg an der Havel, Germany; Qatar University, QATAR

## Abstract

**Objective:**

Stroke remains a leading cause of premature death, impairment and reduced quality of life. Its aftercare is performed by numerous different health care service providers, resulting in a high need for coordination. Personally delivered patient navigation (PN) is a promising approach for managing pathways through health care systems and for improving patient outcomes. Although PN in stroke care is evolving, no summarized information on its cost-effectiveness in stroke survivors is available. Hence, the aim of this systematic review is to analyze the level of evidence on the cost-effectiveness of PN for stroke survivors.

**Methods:**

A systematic literature search without time limitations was carried out in PubMed, EMBASE, CENTRAL, CINAHL as well as PsycINFO and supplemented by a manual search. Randomized controlled trials published prior to April 2020 in English or German were considered eligible if any results regarding the cost-effectiveness of PN for stroke survivors were reported. The review was conducted according to PRISMA guidelines. Quality of included studies was assessed with the RoB2 tool. Main study characteristics and cost-effectiveness results were summarized and discussed.

**Results:**

The search identified 1442 records, and two studies met the inclusion criteria. Quality of included studies was rated moderate and high. Programs, settings and cost-effectiveness results were heterogeneous, with one study showing a 90% probability of being cost-effective at a willingness to pay of $25600 per QALY (health/social care perspective) and the other showing similar QALYs and higher costs.

**Conclusions:**

Since only two studies were eligible, this review reveals a large gap in knowledge regarding the cost-effectiveness of PN for stroke survivors. Furthermore, no conclusive statement about the cost-effectiveness can be made. Future attempts to evaluate PN for stroke survivors are necessary and should also involve cost-effectiveness issues.

## Introduction

Stroke is the second most common cause of death (5.5 million cases annually). Its burden on health care systems remains high, with 13.7 million annual incident cases worldwide [1]. Strokes are mostly associated with psychological, cognitive and physical impairments, which become more onerous with increasing severity [2]. Due to these impairments, the risk of hospital readmission post stroke is high, and the costs of post stroke care can be very high, depending on severity and the length of hospital stay [3, 4]. A stroke affects not only the lives of patients but also the lives of their relatives and caregivers, highlighting the individual burden and the public health relevance of stroke [5, 6].

Thus, stroke survivors need long-term care and support in various areas of life. Care in general and the transition from hospital to outpatient care and rehabilitation in particular present many challenges. These challenges include sufficient information or assistance in accessing health care after hospital discharge (e.g., locating services, scheduling appointments) as well as appropriate education and training regarding the transition to the home environment [7].

One option for enhancing the transition between different care environments and thus improving patient-oriented outcomes such as quality of life is the concept of patient navigation (PN). Initially introduced in the context of barriers to timely breast cancer care for African-American women in Harlem, New York, current PN programs mostly focus on the abolition of one or multiple barriers to accessing appropriate health care services over a wide range of diseases [8–10]. Such programs can be very heterogeneous and be conducted by a variety of usually specifically trained health care professionals or lay health workers [11, 12]. The components of PN programs vary with targeted barriers and can include for example, help in care coordination, emotional support, transportation services, educational modules and financial assistance components [13].

Within the context of stroke, PN is an evolving and promising approach for addressing barriers to appropriate health care and improving patient-oriented outcomes of stroke survivors [14–18]. Among others, PN for stroke survivors is associated with improvements in quality of life, treatment and prevention compliance as well as reduced hospital readmissions.

However, the general cost-effectiveness of such programs has not yet been investigated in a systematic and quantitative way. Therefore, the aim of this systematic review is to quantitatively analyze the scientific literature to assess the level of evidence for randomized controlled trials (RCTs) on the cost-effectiveness of PN programs for stroke survivors compared to a control group.

## Methods

This study was conducted according to Preferred Reporting Items for Systematic Reviews and Meta-Analysis (PRISMA) guidelines and registered in the International Prospective Register of Systematic Reviews (PROSPERO) (registration number CRD42020219088) (see S1 Table) [19].

### Search strategy

The PubMed, EMBASE, CENTRAL, CINAHL and PsycINFO databases were systematically searched for publications prior to April 2020 using predefined keywords and Medical Subject Headings (MeSH) to identify RCTs regarding the cost-effectiveness of PN programs for patients after experiencing a stroke. Broad search term combinations (keywords and MeSH) related to stroke, PN and cost-effectiveness analyses were used. The PubMed search strategy (see S1 Appendix) was adapted to the different databases. The systematic search included the gray literature especially conferences, books and dissertations and was supplemented by a manual screening of the reference lists of key studies, reviews, and study registers to identify additional eligible studies. Furthermore, the authors of study protocols were contacted to retrieve potentially unpublished results. Article inclusion was restricted to

1. RCTs
2. reporting cost-effectiveness results of PN programs for patients with a confirmed stroke,
3. that were written in German or English.

Based on a modification of the cancer-related PN definition by Wells et al. 2008, PN was defined as an intervention that focuses on the detection and elimination of individual barriers to appropriate health care and that is carried out personally (e.g., by care managers, nurses, lay health workers) for a defined episode of stroke-related care [11]. Since this review focuses on stroke survivors, articles were a priori excluded if they solely addressed caregivers or the results were not distinguishable between caregivers and patients. No further restrictions regarding stroke type, the number of previous strokes of participants, reported effectiveness and cost outcomes, and the evaluation perspective were applied. Similarly, no restrictions regarding follow-up lengths, publication date, or participant characteristics (such as age and sex) were used. Articles were excluded if they did not meet the criteria above.

### Study selection and quality assessment

Records from all retrieved search results (systematic and manual) were downloaded and merged with Endnote version X9.2 for Windows (Clarivate Analytics, Boston, MA, USA). In the first step, the titles and abstracts of all retrieved records were screened. In the next step, the full texts were reviewed for potential study inclusion. Data from the included studies were extracted with a standardized spreadsheet (including relevant information on the PN intervention, study design and setting, participants, outcomes, and results). The revised Cochrane tool for assessing risk of bias in randomized trials (RoB2) was used to assess the quality of the included articles [20]. The RoB2 tool assesses the risk of bias of randomized trials in five distinct domains: 1) the randomization process, 2) deviations from the intended interventions, 3) missing outcome data, 4) measurement of the outcome, and 5) selection of the reported results. The tool specification for cluster-randomized trials contains an additional sixth domain, “bias arising from the timing of identification and recruitment of individual participants in relation to timing of randomization”, which was applied if applicable. Each domain contains several signaling questions. The five or six domain judgments lead to an overall risk-of-bias judgment. All domains and the overall judgment can result in “low risk of bias”, “some concerns”, and “high risk of bias”. The study protocols and trial registry records of the included studies were incorporated into the assessment. Uncertainties regarding specific domain questions were tried to resolve through personal contact with the corresponding authors. The RoB2 results were used for descriptive purposes.

The entire screening, data extraction and quality assessment process was independently performed by two authors (B.K. and C.D.). These authors worked closely together with an experienced senior author (T.R.) throughout the review process. Discrepancies regarding article feasibility and risk of bias were discussed between the authors, and consensus was achieved.

## Results

### Study selection

**Fig 1:**
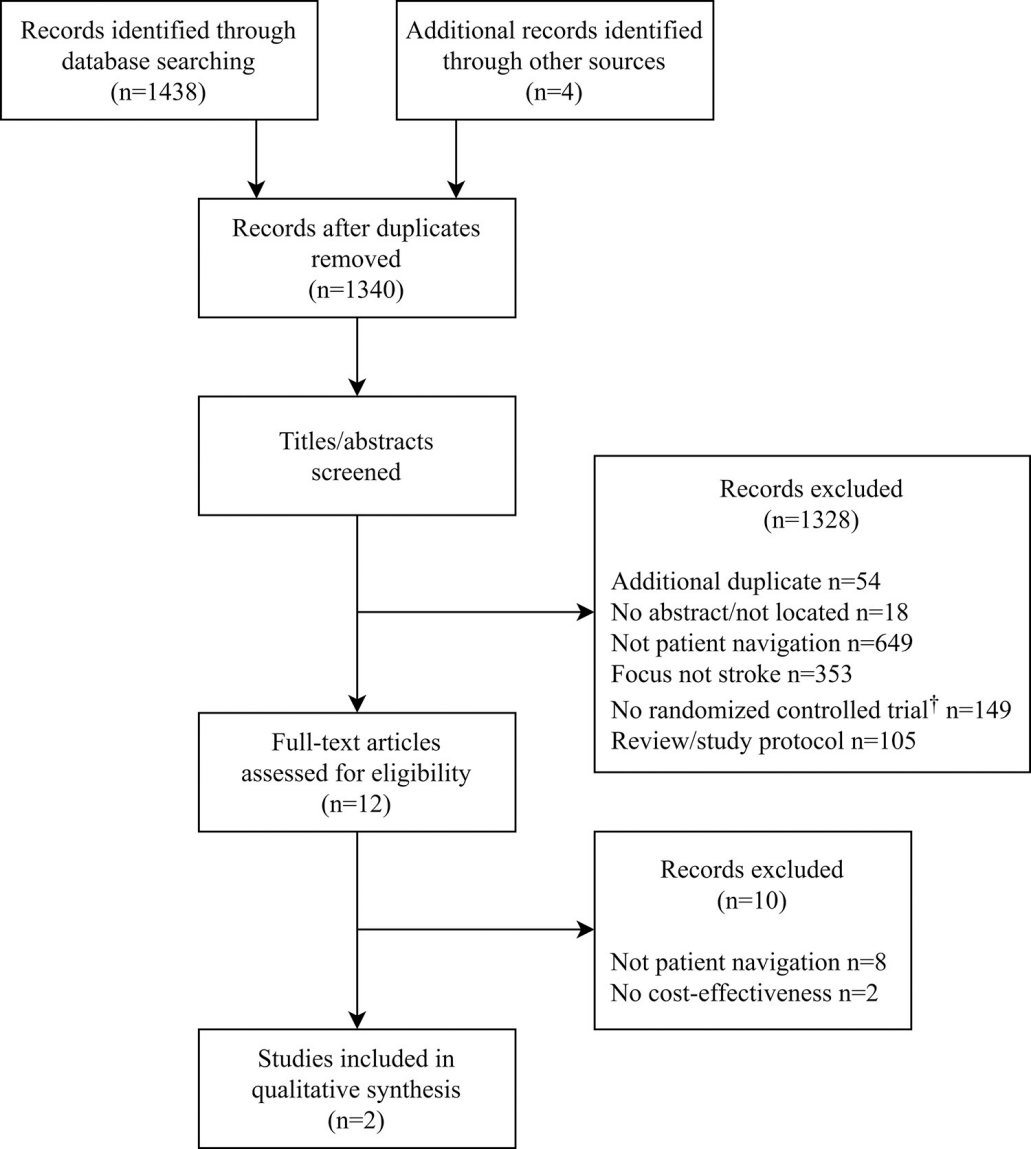
Flowchart of the study identification and selection process. ^†^e.g., policy or legal statements, guidelines, expert opinions, qualitative studies and case studies.

The initial database and manual search identified 1442 records (PubMed n = 960, EMBASE n = 147, CENTRAL n = 208, CINAHL n = 103, PsycINFO n = 20, manual n = 4). After removal of duplicates, 1340 records were screened, and if applicable, abstracts and full texts were reviewed for inclusion. A total of 1338 records did not meet the inclusion criteria. Among these, a focus on something other than stroke (n = 353) and interventions that did not meet the PN definition (n = 657) were the predominant reasons for exclusion. Full text reviews were carried out for 12 records. The PN definition was not met in eight studies [21–29], and two studies did not present cost-effectiveness results [14, 30]. Hence, two studies were included in this review [31, 32]. Fig 1 displays the PRISMA flowchart to illustrate the article identification and selection process. If more than one exclusion criterion was met, the first criterion observed was stated. The interrater reliability between the two reviewers was 100% during the full-text review.

### Study characteristics

Table 1 provides the main characteristics of the two included studies [31, 32]. Both studies were performed in the United Kingdom between 2009 and 2015. The cluster RCT carried out by Forster et al. 2015 collected and analyzed data from 32 stroke care coordinators (SCCs) and 800 participants (intervention group n = 401). The RCT by Rodgers et al. 2019 included 573 participants (intervention group n = 285) newly diagnosed with stroke. Both studies included slightly more men than women, and the participant ages were comparable (see Table 1). Both studies used PN as the primary intervention and incorporated cost-effectiveness analyses. However, the PN programs and settings were different. The study by Forster et al. delivered PN with an evidence-based care system through SCCs compared to usual national health care recommended stroke care coordination in a pragmatic cluster RCT setting with a 12-month follow-up. The intervention focused on the longer-term needs and problems of patients after stroke and of their carers [31]. The PN of the study by Rodgers et al. was nested in an early discharge setting after stroke and compared participants in the intervention group to usual care alone over a 24-month follow-up period. PN was delivered by senior members of an early discharge unit (e.g., physiotherapist, occupational therapist, nurse). Over an 18-month intervention period, regular care was accompanied by continuous interviews to identify patient needs in the context of rehabilitation and goal setting. Action plans were agreed upon and reviewed to maximize participants’ recovery and ability to manage their day-to-day activities.

**Table 1:** Characteristics of the included studies.

Author (year of publication)	Study design & setting	Intervention	Sample	Outcome measures
Forster et al. (2015) United Kingdom	Pragmatic cluster RCT*; participants in the United Kingdom randomly assigned to intervention or control service followed up for 12 months; economic evaluation from both the health/social care and societal perspectives	Longer-term stroke (LoTS) care is an evidence-based system of care delivered by a stroke care coordinator (SCC) that aims to meet the longer-term needs of patients with strokes and their carers living at home. It includes a structured 16-question assessment of patient- and carer-centered problems that is, e.g., linked to a treatment algorithm and transformed into individual care plans for the patients (and, if relevant, their carers), including a dynamic goal and action planner.	N = 800 participants (with confirmed primary diagnosis of new stroke) & 32 SCC services	Primary outcome: Patient-reported psychological well-being according to General Health Questionnaire-12 (GHQ-12) after 6 months.
				Secondary outcomes: *Patient reported* GHQ-12 after 12 months; Frenchay Activities Index, Barthel Index, European Quality of Life-5 Dimensions; Longer-term Unmet Needs after Stroke questionnaire
			Intervention (n = 401; mean n per cluster/SSC service 26.7 (range 2, 45))	
				*Carer reported* GHQ-12; Carer Burden Scale; Client Service Receipt Inventory.
				Economic evaluation: Costs (basis 2011) were linked with the GHQ-12 and quality-adjusted life years (QALYs) at 6 months (primary economic endpoint) and 12 months. Costs were calculated by attaching unit costs to individually self-reported resource use. Cost-effectiveness acceptability curves were constructed (threshold range £0 to £2000 for GHQ-12 point gains and £0 to £50000 for QALY gains) using 5000 bootstrap replications to examine the intervention’s probability of cost-effectiveness.
			Age, mean: 70.9 years (SD 13.2 years)	
			Sex, male, n: 215 (53.6%)	
			Control (n = 399; mean n per cluster/SSC service 28.5 (range 15, 46))	
			Age, mean: 72.5 years (SD 12.8 years)	
			Sex, male, n: 218 (54.6%)	
			268 participants analyzed in the intervention group; 281 analyzed in the control group	
		Controls received usual care (SCC services in accord with existing local policies and practices).		
Rodgers et al. (2019)	Pragmatic parallel-group multicenter observer-blinded RCT with 24 months follow-up in the United Kingdom; economic evaluation from the health/social care perspective	The extended stroke rehabilitation service (EXTRAS) comprised rehabilitation reviews between 1 and 18 months’ post discharge from routine early supported discharge. All reviews included semi structured interviews to reveal rehabilitation issues, goal setting and action planning. Interviews were intended to be conducted via telephone; if they were not possible, home visits were made. Controls received usual care.	N = 573 participants (with new stroke) & 19 UK National Health Service (NHS) study centers.	Primary outcome: Patient-reported performance in extended activities of daily living (NEADL scale) at 24 months post randomization.
				Secondary outcomes: Patient-reported health status (OHS), mood (HAD scale), and experience of services at 12 and 24 months post-randomization
			Intervention (n = 285)	
				Economic Evaluation: Costs (basis 2017) were linked with QALYs (derived from the EQ-5D-5L) over the trial period of 24 months. Costs were calculated using resource utilization data collected with an adaptation of the Client Service Receipt Inventory. The cost-effectiveness plane was constructed using QALY data.
			Age, median: 71 (IQR 60–77)	
			Sex, male, n: 174 (61.1%)	
			Control (n = 288)	
			Age, median: 71 (IQR 62–79)	
			Sex, male, n: 168 (58.3%)	
			235 participants analyzed in the intervention group; 259 analyzed in the control group	

*RCT = randomized controlled trial.

In both studies, costs were determined by means of self-reported resource use questionnaires. Resource consumption was monetarily valued by both research groups by attaching standardized unit costs afterwards. The resulting total costs were calculated from the health and social care perspective over the specific follow-up lengths in both studies, while Forster et al. additionally covered the societal perspective. In the cost-effectiveness analyses, effectiveness was measured in both studies in terms of quality adjusted life years based on generic quality of life questionnaires. The QALY results published by Forster et al. were based on a not further defined version of the EQ-5D questionnaire, while Rodgers et al. applied the EQ-5D-5L questionnaire. For the cost-effectiveness analysis, both studies linked the results on total costs with their QALY findings. Furthermore, Forster et al. linked their total cost results to the findings of the self-reported 12-item General Health Questionnaire (GHQ-12), which was a priori defined as the primary effectiveness outcome.

### Cost-effectiveness results of the included studies

After 12 months, the study by Forster et al. 2015 found similar GHQ-12 values and related QALYs in both groups and additional overall costs for the intervention group, failing to indicate evidence for PN as cost-effective from both perspectives. Rodgers et al. 2019 reported QALY differences of 0.07 (95% CI: 0.01 to 0.12) and cost differences of -$450 (-4908 to 4031) after 24 months both in favor of the intervention group, resulting in a mean net benefit of $2476 at a willingness to pay (WTP) of $28940 for an additional QALY. Considering the same WTP, the authors found a 90% probability of cost-effectiveness for the intervention. More details about the results of the studies are presented in Table 2.

**Table 2:** Results of the included studies.

Author/Country	Effectiveness Results	Cost Results	Cost-effectiveness Results
Forster et al. (2015) United Kingdom (UK)	Patient-reported GHQ-12 differences (control—intervention group) of -0.6 (95% CI: -1.8 to 0.7) after 6 months and 0.5 (95% CI: -0.9 to 2.0) after 12 months.	Total cost differences after 6 months (intervention—control group) of £98 (95% CI: £-721 to £917) [$140* (95% CI: $-1031 to $1311] from the health/social care perspective and £1663 (95% CI: £56 to £3271) [$2378 (95% CI: $80 to $4678] from the societal perspective.	Based on the cost and effectiveness results, incremental cost-effectiveness ratios were not calculated. The results did not indicate cost-effectiveness for the intervention.
	QALY differences (intervention—control group) of -0.04 (95% CI: -0.12 to 0.03) after 6 months and -0.01 (95% CI: -0.03 to 0.01) after 12 months.	Total cost differences after 12 months of £291 (95% CI: £-316 to £898) [$416 (95% CI: $-452 to $1284] from the health/social care perspective and £4135 (95% CI: £-618 to £7652) [$5913 (95% CI: $-884 to $10942] from the societal perspective.	
Rodgers et al. (2019) UK	QALY difference of 0.07 (95% CI: 0.01 to 0.12) in favor of the intervention group after 2 years.	Total cost difference of £311 (-3392 to 2787) [$-450^†^ (95% CI: $-4908 to $4031)] in favor of the intervention group from the health/social care perspective after 2 years.	The probability of being cost-effective at a willingness to pay of £20.000 [$25600] per QALY equals 90% after 2 years.

*UK Sterling (£) was converted to US Dollars ($) using the purchasing power parity rate for 2011 (£1 = $1.43) as proposed by the authors.

^†^Rodgers et al. used the purchasing power parity rate for 2017 (£1 = $1.447).

### Quality assessment

Risk of bias was rated overall “low” for the study by Forster et al., with all domains of the extended RoB2 tool for cluster RCTs rating “low”.

For the study by Rodgers and colleagues, two of the five domains of the RoB2 tool were judged as “some concerns”, resulting in an overall risk of bias judgment of “some concerns”. The main concerns arose from the study protocol deviation regarding the cost-effectiveness results [33]. In contrast to the planned analyses, only QALYs and not the scores of the Nottingham Extended Activities of Daily Living (NEADL) scale at 24 months were linked to total costs. The adjusted mean NEADL scale difference of 1.8 (95% CI: -0.7 to 4.2) in favor of the intervention group was smaller than the minimum clinically important difference on the NEADL scale of 6. Hence, the intervention did not have a favorable effect on patients’ functional status as assessed with the NEADL scale. Only when QALYs were linked to total costs over 24 months did the intervention seem to be cost-effective. The corresponding author was contacted to comment on the topic and referred to the later published full report of the study (see also the discussion) [34]. More information about the risk of bias assessment is displayed in Table 3.

**Table 3:** Domain and global risk of bias assessment by using the revised Cochrane tool for assessing risk of bias in randomized trials.

Domain	Rating	
	Forster et al. 2015	Rogers et al. 2019
Randomization process	low risk	low risk
Bias arising from the timing of identification and recruitment of individual participants in relation to the timing of randomization*	low risk	not applicable
Deviations from the intended interventions	low risk	low risk
Missing outcome data	low risk	low risk
Measurement of the outcome	low risk	some concerns
Selection of the reported results	low risk	some concerns
**Overall rating**	**low risk**	**some concerns**

*Additional domain for cluster randomized trials.

## Discussion

This review aimed to assess and summarize the level of evidence on the cost-effectiveness of PN programs for stroke survivors. To the best of our knowledge, this study is the first systematic review of the cost-effectiveness of PN in the context of stroke. We identified only two studies meeting the inclusion criteria, revealing a large gap in knowledge on the topic. Just like the underlying cost-effectiveness results, the programs were heterogeneous, with one study showing a 90% probability of being cost-effective at a WTP of $25600 per QALY (health/social care perspective) and the other showing similar QALYs and higher costs (both health/social care and societal perspectives). These results, in combination with the small number of included studies, do not allow for a conclusive statement about the overall cost-effectiveness of PN for stroke survivors, and they demonstrate the need for further research in the field.

Our quality assessment of the included studies ranged from moderate (overall RoB2: “some concerns”) to high quality (“low risk of bias”). After our final search was completed, the full report related to the study by Rogers et al. 2019 was published [34]. This report was brought to our attention by the corresponding author. The report included further, hitherto unpublished and less favorable cost-effectiveness results related to the NEADL scale, which was the primary cost-effectiveness outcome according to the study protocol (see the results section). The publication of these results resolved some of our risk of bias concerns. However, we did not change our overall risk of bias rating. In our opinion, these results should have been mentioned in the main publication, and we believe that we correctly scored this publication in line with the RoB2 scoring guide.

Compared to PN for patients with different types of cancer or chronic diseases, the results of the studies included here are not as promising. PN in these fields has been shown to be more beneficial in terms of improving patient-oriented outcomes (e.g., quality of life) and having equal or lower health care costs [9, 11–13]. One explanation could be the heterogeneity in the aftercare needs of stroke survivors and their generally high unmet needs, which vary tremendously on the individual patient level. Inevitably, stroke survivors with severe hemiparesis living in a nursing home have needs that are different from those of stroke survivors with less severe or no spasticity issues living at home. This heterogeneity might lead to difficulties with respect to the construction of PN programs [35, 36]. However, the heterogeneity of PN programs, e.g., in terms of navigation goals and the personal provision of services, and the different courses of disease across the continuum of diseases contradict the comparison across the care continuum.

Following the high standards for systematic reviews and making additional attempts to gather unpublished results were strengths of the present review. However, this review has several limitations that should be noted. The main concern arises from the small number of included studies, which calls into question the generalizability of the results. One reason for the low number of studies meeting our inclusion criteria may be our restriction to RCTs. Since modeling studies, which are often used in health economics research, are susceptible to uncertainty mostly due to the model assumptions, the restriction to RCTs was a priori defined to focus only on the highest level of evidence. Another reason can be found in the language restrictions of the author group, which allowed only studies published in English and German to be included. Nevertheless, we used a broad search strategy which was not restricted to RCTs and included the gray literature. Yet, we were unable to identify further relevant or ongoing studies, even when considering gray literature and additional languages at the level of title screening. Studies that implemented PN but that did not report cost-effectiveness outcomes were excluded. These studies could still contribute to the evaluation of PN; however, this review aimed to assess the cost-effectiveness of PN programs for stroke survivors and not the general effectiveness of PN. Moreover, even though studies meeting our broad definition of PN and our other inclusion criteria could be identified with our search strategy, it is noteworthy that no study labeled its intervention as PN. This finding suggests that the terminology remains uncommon in the context of stroke. Even though the term stroke nurse navigator was introduced more than 10 years ago in the United States and the underlying study showed promising results with respect to increased drug compliance, physician follow-up and potentially decreased rehospitalization rates [17]. Considering the scarcity of the term PN in the context of stroke, highlights the importance of broad search terms, which we applied, to identify the available body of evidence in the field.

Furthermore, both included studies were conducted only in the UK. Since health care resource consumption and costs are influenced by local health care structures (e.g., different payment systems, health provider incentives), the transferability of these results to other countries is limited [37]. In addition, different national health care systems face different degrees of fragmentation [38, 39]. These differences in the degree of fragmentation may also have an influence on PN effectiveness, which might further limit the generalizability of the results. As more providers are involved in patient care, the need for coordination and cooperation is increasing and could be managed by PN.

## Conclusions

The cost-effectiveness of PN for stroke survivors is an underrepresented research field. Only two studies meeting the inclusion criteria could be identified. Thus, our study reveals a large gap in knowledge, and no conclusive statement about the cost-effectiveness of PN programs for stroke survivors can be made. Since one of two studies suggested cost-effectiveness of PN for stroke survivors while the other presented comparable quality of life and cost results in both groups, the research into PN in stroke survivor care should be pursued further to close the revealed gap in knowledge. This could help stakeholders make more informed decisions with respect to the promotion and implementation of PN programs for stroke survivors. Although the implementation of new health care models such as PN depends in part on their cost-effectiveness, future PN studies in regard to stroke survivors are needed and should include comprehensive health economic analysis strategies.

## Supporting information

S1 Table PRISMA 2009 checklist. (DOCX)

S1 Appendix PubMed search strategy. †MeSH, Medical Subject Headings.
(PDF)
