# Supplementary material for: Cost-effectiveness of patient navigation programs for stroke patients–A systematic review
Source: PLoS One. 2021 Oct 15;16(10):e0258582. doi: 10.1371/journal.pone.0258582 (PMC8519430; doi:10.1371/journal.pone.0258582)
Supplement: S1 Appendix — †MeSH, Medical Subject Headings. (PDF) [file pone.0258582.s002.pdf]

Appendix 1. PubMed search strategy.

#1	(((patient navigation [MeSH Terms[†]]) OR navigation) OR navigator) OR patient navigat*) OR nurse navigat*) OR clinical navigat*) OR client navigat*) OR system navigat*) OR professional navigat*) OR navigation program*) OR navigator nurse*) OR navigation service*) OR case management) OR case manager) OR health navigat*) OR health worker) OR social worker) OR case coordinator) OR case coordination) OR health guide) OR community health worker*) OR community navigator*) OR health navigat*) OR stroke navigat*) OR adherence supporter*) OR community health advis*) OR community health advocate*) OR consejer*) OR embajador*) OR health aide*) OR health coach) OR health communicator*) OR health volunteer*) OR lay health advisor*) OR lay health adviser*) OR lay health advocate*) OR lay health educator*) OR lay health promoter*) OR lay health representative*) OR lay health worker*) OR lay navigat*) OR lay outreach worker*) OR community outreach worker*) OR outreach worker*
#2	(((((((((((((((((stroke [MeSH Terms]) OR acute stroke [MeSH Terms]) OR brainstem stroke [MeSH Terms]) OR cerebral stroke [MeSH Terms]) OR cerebrovascular stroke [MeSH Terms]) OR heat stroke [MeSH Terms]) OR lacunar stroke [MeSH Terms]) OR middle cerebral artery stroke [MeSH Terms]) OR posterior cerebral artery stroke [MeSH Terms]) OR stroke) OR cerebrovascular disorders) OR brain ischemia) OR carotid artery diseases) OR (intracranial embolism and thrombosis)) OR intracranial hemorrhage) OR brain infarction) OR vertebral artery dissection) OR apoplexy
#3	((((((((((cost-benefit analysis [MeSH Terms]) OR cost-effectiveness) OR cost-utility analysis) OR cost-utility) OR economic evaluation*) OR marginal analysis) OR cost-benefit) OR cost$) OR cost consequence) OR (costs and cost analysis [MeSH Terms])) OR health care costs
#4	#1 AND #2 AND #3

[†]MeSH, Medical Subject Headings.
